# A2B adenosine receptor activation switches differentiation of bone marrow cells to a CD11c^+^Gr‐1^+^ dendritic cell subset that promotes the Th17 response

**DOI:** 10.1002/iid3.74

**Published:** 2015-07-30

**Authors:** Dongchun Liang, Aijun Zuo, Hui Shao, Mingjiazi Chen, Henry J. Kaplan, Deming Sun

**Affiliations:** ^1^ Department of Ophthalmology of the University of California Los AngelesDoheny Eye InstituteCalifornia90033USA; ^2^ Department of Ophthalmology and Visual SciencesKentucky Lions Eye CenterUniversity of LouisvilleLouisvilleKentucky40202USA

**Keywords:** Adenosine receptors, autoimmunity, experimental autoimmune uveitis, γδ T cells, IL‐17, Th17, uveitis

## Abstract

Adenosine is one of the major molecules associated with inflammation. We have previously reported that an adenosine receptor (AR) agonist has an enhancing effect on Th17 autoimmune responses, even though it suppressed Th1 responses. To determine the mechanism involved, we have examined the effect of AR agonists on mouse bone marrow dendritic cell (BMDC) differentiation and function. We show that mouse bone marrow cells (BMCs) differentiated into CD11c^+^Gr‐1^+^ dentritic cells (DCs) when cultured in granulocyte macrophage colony‐stimulating factor (GM‐CSF)‐containing medium containing an AR agonist. The non‐selective AR agonist NECA and an A2BR‐specific agonist had a similar effect, and the effect of NECA could be blocked by an A2BR‐specific antagonist. Unlike CD11c^+^Gr‐1^−^ BMDCs, which have a greater stimulatory effect on Th1 T cells than Th17 cells, CD11c^+^Gr‐1^+^ BMDCs had a greater stimulatory effect on Th17 autoreactive T cells than on Th1 autoreactive T cells and this effect depended on γδ T cell activation.

## Introduction

Adenosine, an endogenous purine nucleoside modulates a wide range of physiological functions 1, 2 and plays an important role in tumor growth 3, 4, 5, 6, 7 and inflammation 8, 9, 10, 11. Under physiological conditions, only low concentrations of adenosine are present in the extracellular space, but levels increase dramatically under stressful conditions 12. Adenosine accumulates at inflamed sites as the result of release of adenosine triphosphate (ATP) into the extracellular environment and its subsequent dephosphorylation to adenosine diphosphate (ADP) and adenosine monophosphate (AMP), and a terminal reaction converting AMP to adenosine 12, 13. Recent studies have demonstrated that released adenosine also regulates inflammatory and immune responses 10, 14, 15. Moreover, it can have either a negative 4, 10, 16, 17, 18 or positive 9, 19, 20 effect on these responses by binding to the four different types of AR, designated A1R, A2AR, A2BR, and A3R 14, 17, 21. The general consensus is that activation of A2AR suppresses responses 22, 23, 24, whereas A2BR activation enhances them 20, 25, 26.

Our laboratory is interested in determining (i) the mechanisms by which pathogenic Th17 (IL‐17^+^) and Th1 (IFN‐γ^+^) autoreactive T cells cause autoimmune disease, (ii) the immune factors that are important for Th17 activation, and (iii) whether regulation of the Th17 response differs from that of the Th1 response. We have previously shown that an A2AR agonist inhibits Th1 responses, but can have either an inhibitory or stimulatory effect on Th17 responses 27. To clarify the mechanism by which an AR agonist regulates the immune response and determine the immune cells that are involved in the regulation, we have now examined the effect of AR agonists on mouse dendritic cell (DC) differentiation and function. Our results showed that, when cultured in granulocyte macrophage colony‐stimulating factor (GM‐CSF)‐containing medium, the majority of mouse bone marrow cells differentiated into CD11c^+^Gr‐1^−^ DCs, but when the culture medium also contained the non‐selective AR agonist 50‐N‐ethylcarboxamidoadenosine (NECA) or an A2BR agonist (BAY 60‐6538), a large proportion of the differentiated DCs were CD11c^+^Gr‐1^+^. A functional study showed that CD11c^+^Gr‐1^+^ DCs have a strong stimulatory effect on Th17 autoreactive T cells and γδ T cells, in sharp contrast to CD11c^+^Gr‐1^−^ DCs that preferentially stimulate Th1 cells.

We have recently reported that γδ T cells have a strong regulatory effect on Th17 autoimmune responses and that an increased autoimmune Th17 response is associated with increased activation of γδ T cells 28, 29, 30, 31, 32. To understand the mechanisms by which γδ T cells regulate Th17 responses, we sought to identify molecules that cause γδ T cell activation in vivo. In a previous report 33, we showed that injection of an A2AR agonist during autoimmune inflammation increases the stimulatory effect of γδ T cells on the Th17 response. In the present study, we show that an A2BR agonist has a strong effect on DC differentiation and tips the balance from the generation of DCs that stimulate Th1 responses to those that stimulate Th17 responses and that this regulatory effect involves γδ T cell activation. We conclude that accumulation of extracellular adenosine in an inflammatory environment favors Th17 responses and that modulation of the immune response might be achieved by acting on AR activation or, alternatively, DC differentiation and γδ T cell activation.

## Materials and Methods

### Animals and reagents

Female C57BL/6 (B6) and TCR‐δ^−/−^ mice on the B6 background, purchased from Jackson Laboratory (Bar Harbor, ME), were housed and maintained in the animal facilities of the University of Southern California. All animal studies conformed to the Association for Research in Vision and Ophthalmology statement on the use of animals in Ophthalmic and Vision Research. Institutional approval was obtained from the Institutional Animal Care and Use Committee (IACUC) of the Doheny Eye Institute, University of Southern California, and institutional guidelines regarding animal experimentation were followed.

Recombinant murine IL‐12 and IL‐23 were purchased from R & D (Minneapolis, MN). Fluorescein isothiocyanate (FITC)‐ or phycoerythrin (PE)‐conjugated antibodies against the mouse αβ T cell receptor (αβ TCR), γδ TCR, IL‐17, IFNγ, Gr‐1 (Ly6G/C; clone RB6‐8C5), CD11b (clone M1/70), CD11c (clone N418), CD3 (clone 145‐2C11), or CD69, and isotype control antibodies were purchased from e‐Bioscience (San Diego, CA). The non‐selective AR agonist 50‐N‐ethylcarboxamidoadenosine (NECA), A1R‐specific agonist 2‐chloro‐N6‐cyclopentyladenosine (CCPA), A2AR‐specific agonist 2‐p‐(2‐carboxyethyl) phenethylamino‐5′‐N‐ethylcarboxamidoadenosine (CGS21680), A2BR‐specific agonist 2‐[6‐amino‐3,5‐dicyano‐4‐[4‐(cyclopropylmethoxy) phenyl]pyridin‐2‐ylsulfanyl] acetamide (BAY 60‐6538), A3R‐specific agonist 2‐Cl‐IB‐MECA, A1R‐specific antagonist 8‐cyclopentyl‐1,3‐dipropylxanthine (DPCPX), A2AR‐specific antagonist 7‐(2‐phenylethyl)‐5‐amino‐2‐(2‐furyl)‐pyrazolo‐[4,3‐e]‐1,2,4‐triazolo[1,5‐c]pyrimidine (SCH 58261), A2BR‐specific antagonist N‐(4‐cyanophenyl)‐2‐[4‐(2,3,6,7‐tetrahydro‐2,6‐dioxo‐1,3‐dipropyl‐1H‐purin‐8‐yl) phenoxy]‐acetamide (MRS 1754), and A3R‐specific antagonist MRS1220 were purchased from Sigma–Aldrich (St. Louis, MO).

### Generation of bone marrow dendritic cells

Mouse bone marrow dendritic cells (BMDCs) were generated by incubation of bone marrow cells (BMCs) in the presence of 10 ng/mL of GM‐CSF for 5 days, as described previously 34. BMCs from the femur and tibia of immunized B6 mice were harvested under sterile conditions and 2 × 10^6^ cells were seeded into each well of a 24 well cell culture plate and cultured in complete medium [RPMI 1640 medium (Corning, Manassas, VA) containing 10% fetal calf serum (FCS) (Hyclone, Logan, Utah)] and 10 ng/mL of recombinant murine GM‐CSF (R&D Systems). In studies testing the effect of the presence of adenosine analogs during the 5 day incubation with GM‐CSF, BMCs were incubated with GM‐CSF plus NECA (100 nM), the A1R agonist CCPA (50 nM), the A2AR agonist CGS21680 (250 nM), the A3R agonist 2‐Cl‐IB‐MECA (100 nM), or the A2BR agonist BAY 60‐6538 (100 nM) or NECA with or without the A1R antagonist DPCPX (50 nM), the A2AR antagonist SCH58261(100 nM), the A2BR antagonist MRS1745 (100 nM), or the A3R antagonist MRS1220 (5 µM), then the attached cells were suspended for immune staining and FACs analysis or functional assay.

### Immunization

Experimental autoimmune uveitis (EAU) was induced in B6 mice by subcutaneous injection of 200 µl of emulsion containing 200 μg of the human interphotoreceptor retinoid‐binding protein (IRBP) peptide IRBP_1–20_ (Sigma–Aldrich) in complete Freund's adjuvant (Difco, Detroit) at six spots at the tail base and on the flank and intraperitoneal (i.p.) injection with 300 ng of pertussis toxin, as described previously 28, 30, 32.

### T cell preparation

T cells were purified from the spleens or draining lymph nodes of IRBP_1–20_‐immunized mice by positive selection using a combination of FITC‐conjugated anti‐CD3 antibody and anti‐FITC antibody‐coated Microbeads, followed by separation using an autoMACS separator system according to the manufacturer's suggested protocol (Miltenyi Biotec, Auburn, CA). The purity of the isolated cells, determined by flow cytometric analysis using PE‐conjugated antibodies against αβ or γδ T cells, was >95%.

### Assessment of Th1 and Th17 proliferative responses

A proliferation assay was performed by culturing purified CD3 cells (3 × 10^6^) from IRBP_1–20_‐immunized B6 mice at 37°C for 48 h in 96‐well microtiter plates in complete medium containing graded doses of the immunizing peptide in the presence of BMDCs (1.5 × 10^5^/well) under Th1 polarizing conditions (culture medium supplemented with 10 ng/mL of IL‐12) or Th17 polarizing conditions (culture medium supplemented with 10 ng/mL of IL‐23) in a total volume of 200 µL. [3H] thymidine incorporation during the last 8 h was assessed using a microplate scintillation counter (Packard). The proliferative response was expressed as the mean cpm ± standard deviation (SD) of triplicate determinations.

### Cytoplasmic staining

After 5 days' culture of in vivo primed T cells with the immunizing antigen and APCs under Th1‐ or Th17 polarizing conditions, activated T cells were separated using Ficoll gradient centrifugation and stimulated in vitro for 4 h with 50 ng/mL of phorbol myristic acetate, 1 μg/mL of ionomycin, and 1 μg/mL of brefeldin A (all from Sigma, St. Louis, MO). The cells were then fixed, permeabilized overnight with Cytofix/Cytoperm buffer (eBioscience, San Diego, CA), intracellularly stained with antibodies against IFN‐γ or IL‐17, and analyzed on a FACScalibur.

### Immunofluorescence flow cytometry

Aliquots of 2 × 10^5^ cells were double‐stained with combinations of FITC‐ or PE‐conjugated monoclonal antibodies. Data collection and analysis were performed on a FACS_calibur_ flow cytometer using CellQuest software.

### CFSE assay

Purified CD3^+^ T cells from IRBP_1–20_‐immunized B6 mice were stained with CFSE (Sigma–Aldrich) as described previously 35. Briefly, the cells were washed and suspended as 50 × 10^6^ cells/mL in serum‐free RPMI 1640 medium, then were incubated at 37°C for 10 min with gentle shaking with a final concentration of 5 µM CFSE before being washed twice with, and suspended in, complete medium, stimulated with immunizing peptide in the presence of irradiated syngeneic spleen cells as APCs, and analyzed by flow cytometry.

### Adenosine binding assay

BMCs or in vitro cultured BMDCs were seeded in 96‐well cell culture plates at a density of 1×10^5^/mL in 100 μL of complete medium were incubated at 37°C for 1 h with H^3^‐adenosine at final concentrations of 1.0 μM in triplicate, with or without selective A2BR antagonist MRS 1754 (1 μM), then cell‐bound and free H^3^‐adenosine were separated by harvesting the cells on a cell harvester (Perkin Elmer) and the cell‐associated radioactivity measured by liquid scintillation.

### Statistical analysis

The results in the figures are those from a representative experiment, which was repeated 3–5 times. The statistical significance of differences between groups in a single experiment was initially analyzed by ANOVA, and if a statistical significance was detected, the Student–Newman–Keuls post‐hoc test was subsequently used. A *P*‐value less than 0.01 is indicated as **.

## Results

### BMDCs generated by incubation of BMCs with GM‐CDF plus an AR agonist have an increased ability to stimulate Th17 autoreactive T cells

Our previous studies showed that the A2AR agonist GS 1680 inhibits a Th1 response, but can have an enhancing effect on a Th17 response 27, 33. To determine why its effect on Th1 and Th17 responses differed and how the enhancing effect on Th17 response was generated, we studied its effect on αβ and γδ T cell activation and found that it inhibited αβ T cell activation, but enhanced γδ T cell activation, and inhibited the Th1 autoreactive T cell response, but either inhibited or enhanced the Th17 response 27, 33. Since DCs are important cells in T cell activation, in this study, we examined the effect of various AR agonists on the differentiation and function of BMDCs.

**Figure 1:**
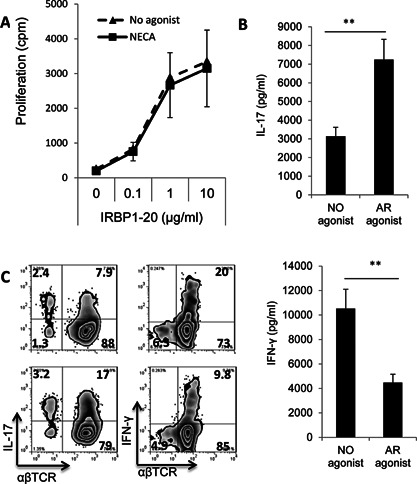
Differentiation of bone marrow cells into BMDCs in the presence of the non‐specific AR agonist NECA results in increased Th17‐stimulating ability. A: Stimulatory effect of mouse BMDCs under non‐polarizing conditions. Bone marrow cells were cultured for 5 days in medium continuing GM‐CSF (10 ng/mL) in the absence or presence of NECA (100 nM), then were detached, washed, and seeded (5 × 10^4^/well) into 24‐well plates Responder CD3 T cells, isolated from immunized B6 mice (10^6^ cells/well) were added to the plates (T cell/DC ratio 20:1), then the cells were incubated in the presence of graded doses of the immunizing peptide for 48 h and T cell proliferation was assessed by ^3^H‐thymidine incorporation. The results shown are the mean ± SD for one study doing in triplicated wells and the experiment was repeated 3 times with similar results. B: Stimulatory effect of mouse BMDCs on Th1 and Th17 autoreactive T cells under polarizing conditions. Responder T cells were co‐cultured for 48 h with each of the two DC preparations (generated in the absence or presence of NECA) and the immunizing peptide under polarizing conditions favoring Th1 cell proliferation (culture medium containing IL‐12) or Th17 cell proliferation (culture medium containing IL‐23), then the culture supernatants were assayed for IL‐17 (top panel) or IFN‐γ (bottom panel). C: Intracellular staining of the proliferating T cells for IL‐17 or IFN‐γ expression. The activated T cells generated in (B) using DCs generated in the absence (top panels) or presence of NECA (bottom panels) were separated on day 2 and cultured for 3 days, then the separated, activated T cells were treated for 4 h with 50 ng/mL of phorbol myristic acetate, 1 μg/mL of ionomycin, and 1 μg/mL of brefeldin A, fixed, permeabilized overnight with Cytofix/Cytoperm buffer, and stained intracellularly with antibodies against IL‐17 (left panels) or IFN‐γ (right panels) and analyzed on a FACScalibur.

To determine whether an AR agonist altered mouse BMDC differentiation, BMCs isolated from B6 mice immunized with the uveitogenic peptide IRBP_1–20_ were cultured for 5 days in medium containing only GM‐CSF (10 ng/mL) or GM‐CSF plus the non‐specific AR agonist NECA (100 nM), then the cells generated were tested for their ability to act as antigen‐presenting cells (APCs) to stimulate the in vitro activation of responder CD3^+^ T cells separated from B6 mice immunized with IRBP_1–20_ (T cell/DC ratio 20:1). An antigen dose–dependent proliferation assay showed similar proliferation of T cells when either set of DCs was used as APCs (Fig. 1A). To determine whether the ability of the two sets of DCs to stimulate Th1 or Th17 autoreactive T cells differed, responder T cells were co‐cultured for 48 h with each of the two DC sets and the immunizing peptide under polarizing conditions favoring proliferation of either Th1 cells (culture medium containing IL‐12) or Th17 cells (culture medium containing IL‐23), then the culture supernatants were collected and assayed for IL‐17 or IFN‐γ. Figure 1B shows that IL‐17 production under Th1 polarizing conditions (top panel) was increased using DCs generated in the presence of NECA, while IFN‐γ production under Th17 polarizing conditions (bottom panel) was decreased. The proliferating T cells were also separated and intracellularly stained with anti‐IFN‐γ or anti‐IL‐17 antibodies, followed by FACS analysis (Fig. 1C; top panels, no NECA; bottom panels, with NECA) and the results showed that DCs generated in the presence of NECA (bottom panels) had a greater stimulatory effect on IL‐17^+^ autoreactive T cell activation than DCs conventionally generated in the absence of agonist (top panels) and that the opposite effect was seen for the stimulation of IFN‐γ^+^ autoreactive T cells.

### AR activation induces BMCs to differentiate into a novel DC subtype co‐expressing CD11c and Gr‐1

**Figure 2:**
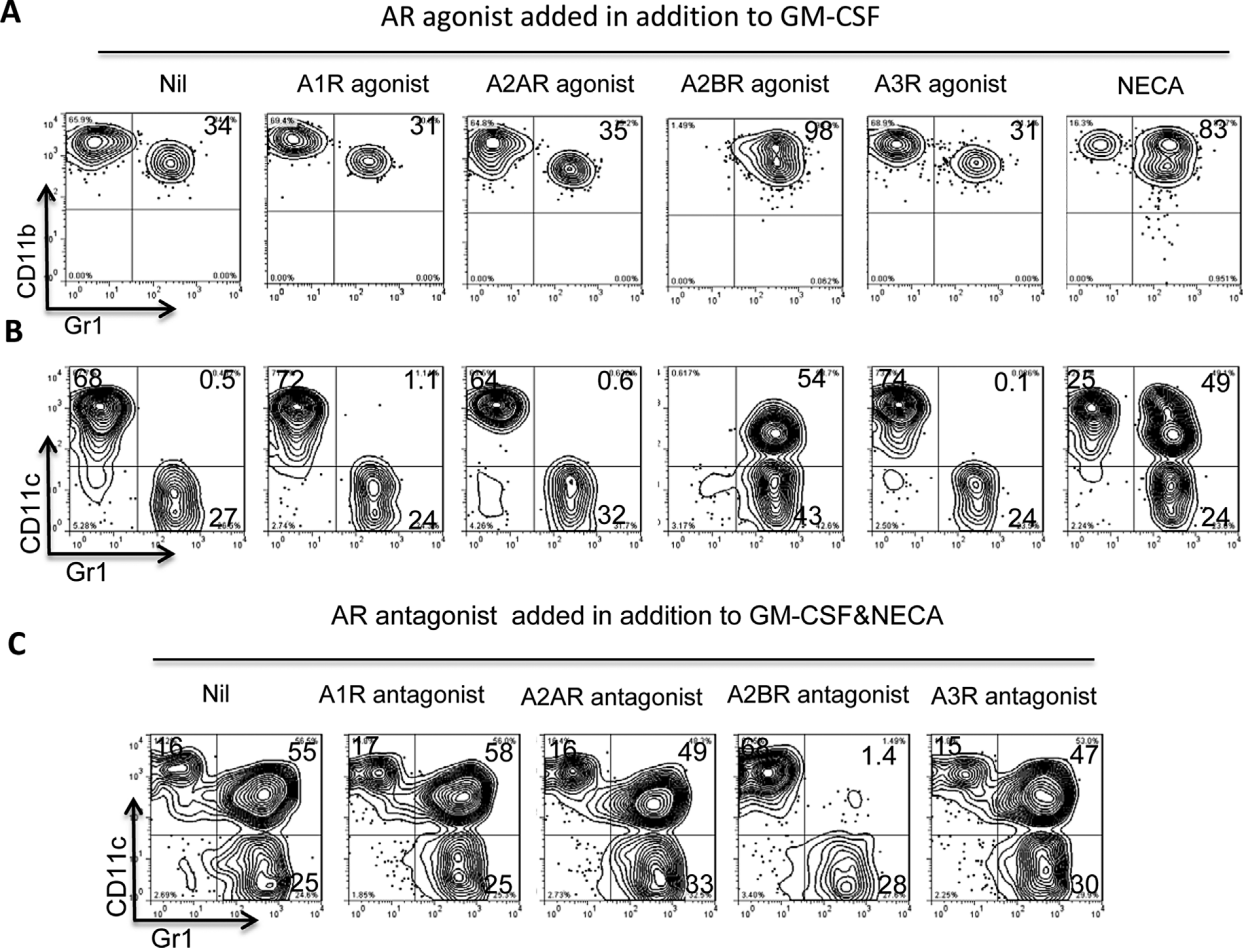
AR activation promotes bone marrow cells to differentiate into a novel DC subtype co‐expressing CD11c and Gr‐1. Mouse bone marrow cells cultured for 5 days in medium containing GM‐CSF (10 ng/mL) in the absence or presence of the indicated AR agonists/antagonist were stained with PE‐conjugated anti‐CD11b or anti‐CD11c antibodies and FITC‐conjugated anti‐Gr‐1 antibodies, then were examined by FACS analysis. A and B: Treatment of in vitro cultured BM cells with the A2BR agonist BAY 60‐6538 (100 nM), but not the A1R agonist CCPA (50 nM), the A2AR agonist CGS 21,680 (250 nM), or the A3R agonist 2‐Cl‐IB‐MECA (100 nM), promotes the differentiation of CD11c^+^Gr‐1^+^ BMDCs. C: The effect of the nonselective AR agonist NECA (100 nM) is blocked by the A2BR antagonist MRS 1754 (100 nM), but not the A1R antagonist DPCPX (50 nM), the A2AR antagonist SCH 58,261 (100 nM), or the A3R antagonist MRS1220 (5 µM). The results shown are from one representative experiment, which was repeated more than five times with similar results.

We then examined how AR activation affected BMDC function. BMCs were incubated for 5 days with GM‐CSF (10 ng/mL) in the presence or absence of various AR agonists or antagonists, then the BMDCs generated were stained with antibodies against mouse CD11b and Gr‐1 and FACS analysis was performed. As shown in Figure 2A, most, if not all, of the proliferating cells in all cultures expressed CD11b, and of those generated in GM‐CSF alone, 34% co‐expressed Gr‐1, while this percentage rose to 98% in the presence of the A2BR‐specific agonist BAY 60‐6538, but not the A1R‐specific agonist CCPA, the A2AR‐specific agonist CGS 21680, or the A3R‐specific agonist (2‐Cl‐IB‐MECA). As shown in Figure 2B, staining with a combination of anti‐CD11c and anti‐Gr‐1 antibodies showed that only a very few cells co‐expressing CD11c and Gr‐1 were generated in medium containing GM‐CSF alone (0.5%) or together with the A1R agonist (1.1%) or the A2AR agonist (0.6%), or the A3R agonist (0.1%), but this number was increased dramatically (54%) when the A2BR agonist was present. As shown in Figure 2C, the non‐specific AR agonist NECA had a similar effect, which was completely blocked by the A2BR antagonist MRS 1745, but not the A1R antagonist DPCPX, the A2AR antagonist SCH 58261, or the A3R antagonist MRS1220, suggesting that the effect resulted mainly from binding of NECA to the A2BR.

### Functional comparison of the separated DC subsets

**Figure 3:**
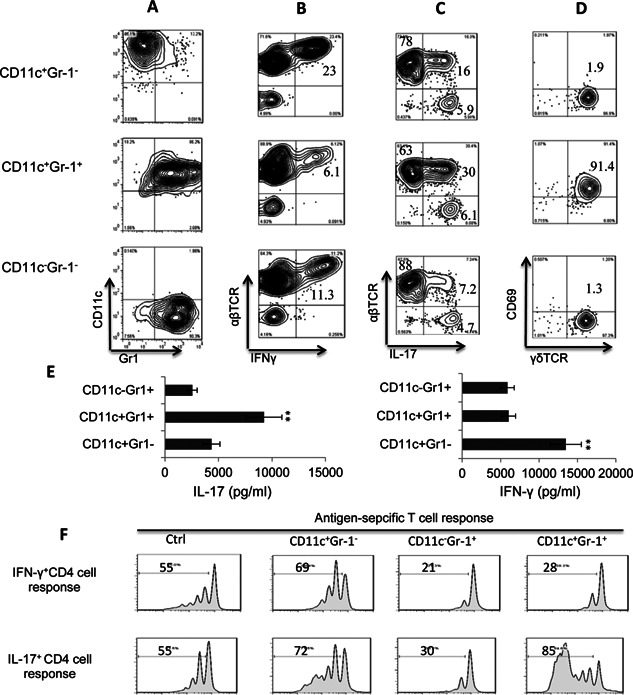
Functional comparison of separated DC subsets. A: Phenotypes of the separated CD11c^+^Gr‐1^−^, CD11c^+^Gr‐1^+^, and CD11c^−^Gr‐1^+^ BMDC subsets. B: Comparison of the ability of the CD11c^+^Gr‐1^−^, CD11c^+^Gr‐1^+^, and CD11c^−^Gr‐1^+^ BMDC subsets to stimulate the generation of IFN‐γ^+^‐autoreactive T cells. Responder T cells from immunized B6 mice were co‐cultured for 5 days with individual BMDC subsets (T cell/DC ratio 20:1) in the presence of the immunizing peptide (IRBP_1–20_) under Th1 polarizing conditions (culture medium containing 10 ng/mL of IL‐12), then the percentage of IFN‐γ^+^‐autoreactive T cells was determined by staining with the indicated antibodies and FACs analysis. C: Comparison of the ability of the CD11c^+^Gr‐1^−^, CD11c^+^Gr‐1^+^, and CD11c^−^Gr‐1^+^ BMDC subsets to stimulate the generation of IL‐17^+^‐autoreactive T cells. The system used was the same as that in (B) except Th17 polarizing conditions were used (culture medium containing 10 ng/mL of IL‐23). D: Comparison of the ability of the CD11c^+^Gr‐1^−^, CD11c^+^Gr‐1^+^, and CD11c^−^Gr‐1^+^ BMDC subsets to stimulate activation of γδ T cells. Responder γδ T cells were isolated from immunized mice and cultured for 5–7 days in cytokine‐free medium, as described previously 28, 30, 32, then were added (1 × 10^5^/well) to cultures pre‐seeded with BMDCs (T cell/DC ratio 10:1) for 3 days, then the T cells were separated and stained with antibodies against the T cell activation marker CD69 and the γδ TCR. E: ELISA assay testing the Th1‐ and Th17 stimulating effect of the CD11c^+^Gr‐1^−^, CD11c^+^Gr‐1^+^, and CD11c^−^Gr‐1^+^ BMDC subsets. Purified CD3 T cells from IRBP1‐20‐immunized B6 mice were incubated with immunizing peptide in the presence of indicated BMDC subset (T cell/DC ratio 10:1) under Th17 (left panels) or Th1 (right panels) polarizing conditions for 48 h, then the culture supernatants were assess for IL‐17 or IFN‐γ. F: CFSE assay testing the suppressive effect of the CD11c^+^Gr‐1^−^, CD11c^+^Gr‐1^+^, and CD11c^−^Gr‐1^+^ BMDC subsets. Purified CD3 T cells from IRBP_1–20_‐immunized B6 mice were stained with CFSE (Sigma–Aldrich), washed, and incubated with immunizing peptide in the presence of irradiated splenic APCs and the indicated BMDC subset (T cell/DC ratio 10:1) under Th1 (top panels) or Th17 (bottom panels) polarizing conditions for 5 days, then the cells were harvested and analyzed by FACS.

Since cultured BMDCs are mixtures of phenotypically distinct cells consisting of the two previously known subsets CD11c^+^Gr‐1^−^ and CD11c^−^Gr‐1^+^ and the CD11c^+^Gr‐1^+^ subset identified in this study, we examined the function of these different DC subsets after separation using a MACS separating column. As shown in Figure 3A, the purity of the separated cells was 85–90%. We then assessed the separated cells for stimulatory activity on Th1 (3B) or Th17 (3C) autoreactive T cells and γδ T cells (3D). The separated DCs were co‐cultured for 5 days with in vitro primed responder T cells derived from immunized B6 mice (T cell/DC ratio 20:1) in the presence of the immunizing peptide IRBP_1–20_ under Th1 polarizing conditions generating IFN‐γ^+^ responder cells or Th17 polarizing conditions generating IL‐17^+^ responder T cells. The produced cytokines were assessed (Fig. 3E) and the activated T cells were separated and cytoplasmic levels of IFN‐γ or IL‐17 examined. Figure 3B shows that a larger percentage of IFN‐γ^+^ T cells were activated when responder T cells were incubated with CD11c^+^Gr‐1^−^ DCs (23%, top panel) than when incubated with CD11c^+^Gr‐1^+^ DCs (6.1%, center panel) or CD11c^−^Gr‐1^−^ cells (11.3%, bottom panel). However, when the generation of Th17 cells was monitored (Fig. 3C), the CD11c^+^Gr‐1^+^ DCs had the strongest stimulatory effect (30% compared to 16% and 7.2%). We then examine the stimulatory effect of these DC subsets on responder γδ T cells isolated from immunized mice and rested by cultured in cytokine‐free medium for 5–7 days 28, 30, 32. These γδ T cells were then added (1 × 10^5^/well) to cultures pre‐seeded with BMDCs (T cell/BMDC ratio 20:1) for 3 days, then the T cells were separated and stained with an antibody against the T cell activation marker CD69. As shown in Figure 3D, CD11c^+^Gr‐1^+^ DCs, but not CD11c^+^Gr‐1^−^ or CD11c^−^Gr‐1^+^ DCs, had a strong stimulatory effect on the expression of CD69 by γδ T cells (91.4% compared to 1.9% or 1.3%). Finally, we compared the suppressive effect of BMDCs on the proliferation of T cells by incubating CSFE‐labeled responder T cells from immunized B6 mice with the antigen and splenic APCs in the absence or presence of the DC subsets (T cell/DC ratio 10:1). Figure 3F shows that addition of CD11c^+^Gr‐1^+^ DCs reduced the Th1 response and increased the Th17 response; whereas addition of CD11c^+^Gr‐1^−^ DCs slightly enhanced both the Th1 and Th17 responses and addition of CD11c^−^Gr‐1^+^ DCs significantly reduced both responses.

### The enhancing effect of CD11c^+^Gr‐1^+^ DCs on the Th17 response requires γδ T cells

**Figure 4:**
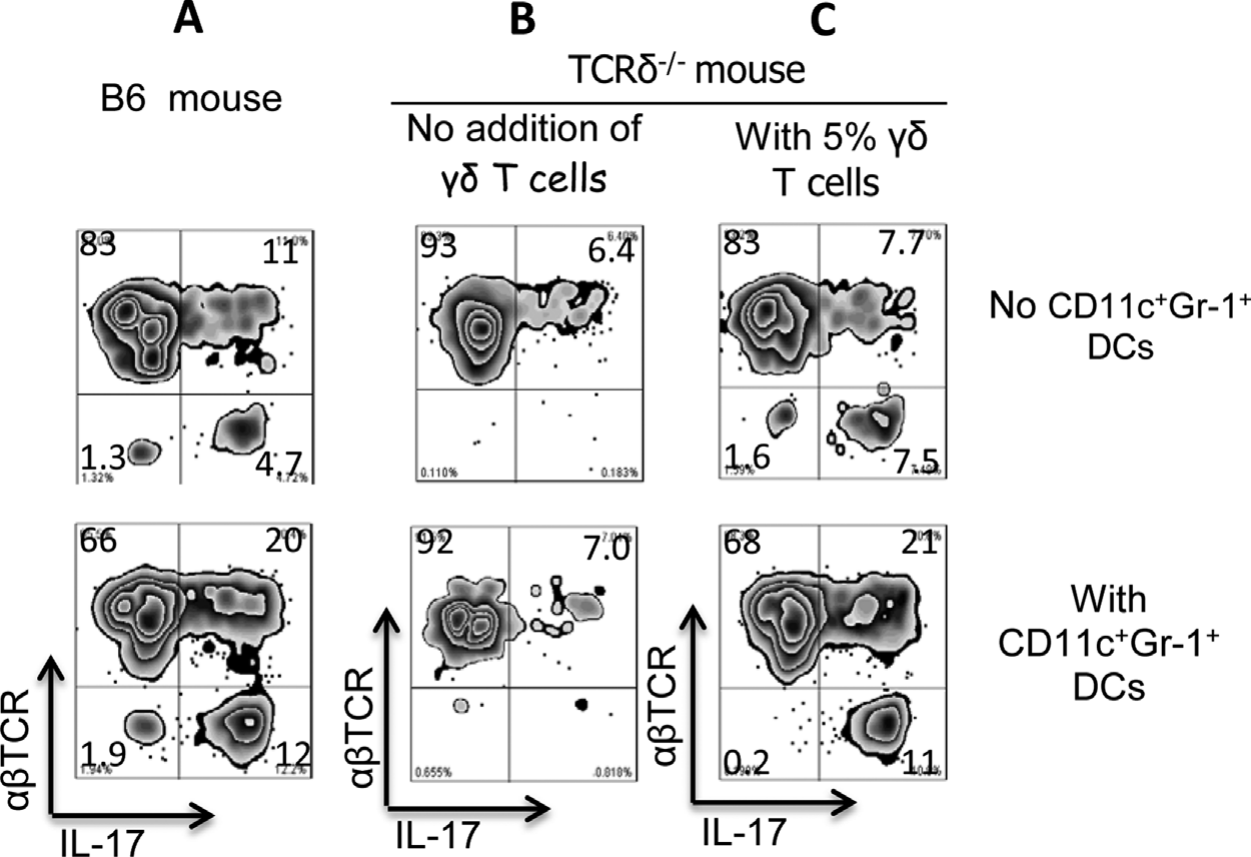
The Th17‐enhancing effect of the CD11c^+^ Gr‐1^+^ BMDC subset requires γδ T cells. A and B: CD11c^+^Gr‐1^+^ BMDCs enhance the activation of IL‐17^+^ autoreactive T cells from B6 mice (A), but not TCR‐δ^−/−^ mice (B). IRBP_1–20_‐specific T cells from immunized B6 or TCR‐δ^−/−^ mice were incubated in vitro under Th17 polarizing conditions with the immunizing peptide and an optimal number of splenic APCs in the absence (top panels) or presence (bottom panels) of CD11c^+^Gr‐1^+^ BMDCs (T cell/DC 10:1), then activation of IL‐17^+^ autoreactive T cells was assessed by estimation of the number of cells stained for IL‐17^+^. B and C: Addition of γδ T cells (5% of total responder T cells) to TCR‐δ^−/−^ responder T cells restores the Th17‐enhancing effect of CD11c^+^Gr‐1^+^ BMDCs. Responder T cells from immunized TCR‐δ^−/−^ mice were incubated in vitro for 5 days with antigen and splenic APCs in the absence (left panels, B) or presence (right panels, C) of γδ T cells with (lower panels) or without (upper panels) addition of CD11c^+^Gr‐1^+^ BMDCs (T cell/DC 10:1), then the proliferating cells were separated, stained with anti‐IL‐17 and anti‐αβTCR antibodies, and analyzed by FACS.

We have previously reported that γδ T cells play a major role in modulating the Th17 autoimmune response 28, 30, 32, 36 and in shaping the effect of an AR agonist on the autoimmune response 27, 33. To examine whether the enhancing effect of CD11c^+^Gr‐1^+^ DCs on the Th17 response required γδ T cells, we compared their effect on Th17 responses in B6 and TCR‐δ^−/−^ mice, which lack functional γδ T cells. The responder T cells isolated from immunized TCR‐δ^−/−^ and B6 mice were stimulated in vitro with the immunizing peptide IRBP_1–20_ and splenic APCs under Th17 polarizing conditions with or without addition of CD11c^+^Gr‐1^+^ BMDCs (T cell/DC ratio 10:1) and activation of responder T cells was monitored by intracellular cytokine expression. The results shown in Figure 4A (B6 mice) and Figure 4B (TCR‐δ^−/−^ mice) demonstrate that addition of CD11c^+^Gr‐1^+^ DCs enhanced the Th17 response of B6 responder T cells, but not TCR‐δ^−/−^ responder T cells. However, addition of 5% exogenous γδ T cells from immunized B6 mice to TCR‐δ^−/−^ responder T cells restored the enhancing effect (Fig. 4C), showing that the enhancing effect of CD11c^+^Gr‐1^+^ DCs on Th17 responses requires γδ T cells.

**Figure 5:**
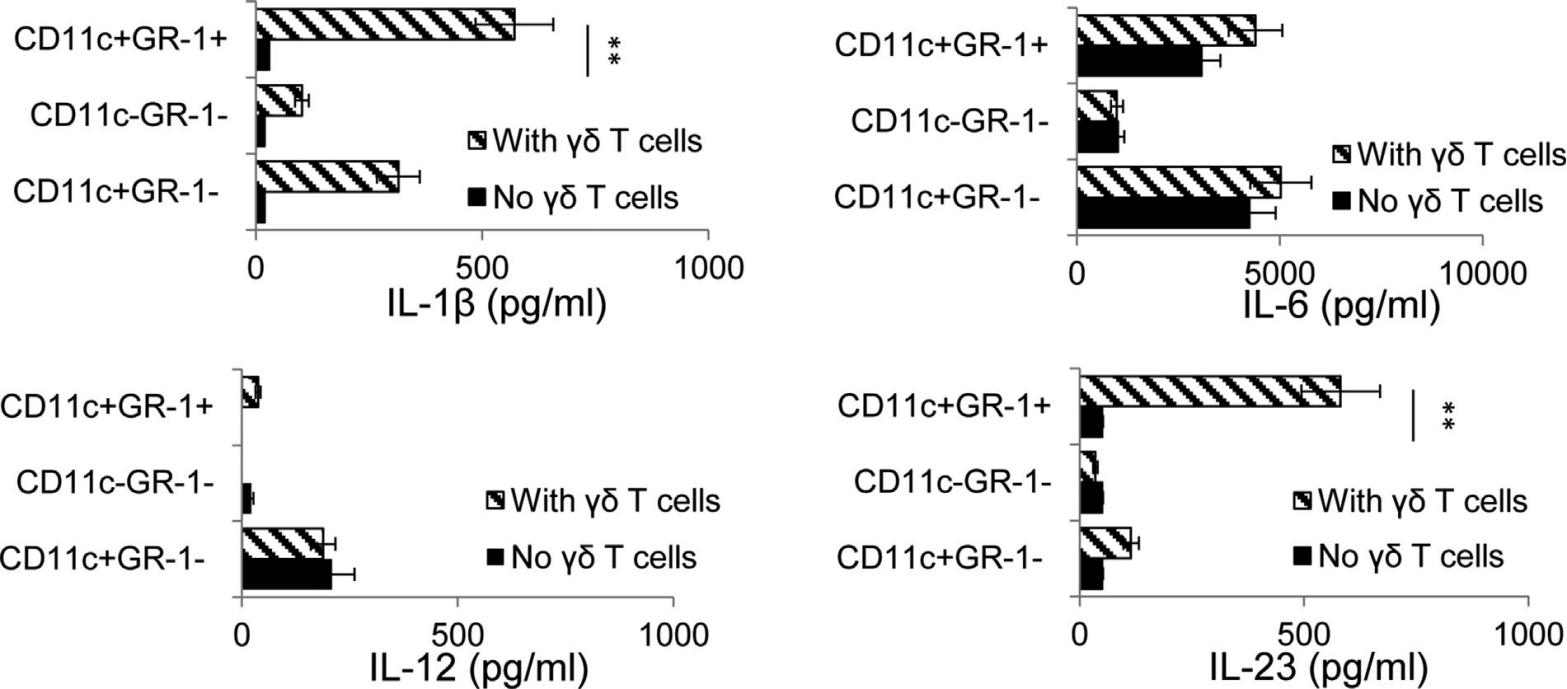
CD11c ^+^ Gr‐1^+^ DCs produce increased amounts of IL‐23 and IL‐1β after co‐culture with γδ T cells. Separated CD11c^+^Gr‐1^−^, CD11c^+^Gr‐1^+^, and CD11c^−^Gr‐1^+^ BMDCs were cultured in 48‐well plates (1 × 10^5^/well) with or without addition of γδ T cells (T cell/DC ratio 1:1) for 48 h, then cytokine levels in the supernatants were assessed using ELISA. The results are the mean ± SD for one study and the experiment was repeated three times with similar results. **P < 0.01.

To determine the mechanism by which CD11c^+^Gr‐1^+^ DCs enhanced the Th17 T cell response and why this enhancing effect required the assistance of γδ T cells, we assessed release of IL‐1β, IL‐6, IL‐12, and IL‐23 into the culture medium by the separated BMDC subsets before and after co‐culture with γδ T cells (γδ T cells/DC ratio 1:1). As shown in Figure 5, the cytokine‐producing activity of the CD11c^+^Gr‐1^−^, CD11c^+^Gr‐1^+^, and CD11c^−^Gr‐1^+^ BMDC subsets differed significantly. When cultured alone, none of the subsets produced significant amounts of the tested cytokines, except IL‐6. However, after co‐culture with γδ T cells, significantly increased amounts of both IL‐1β and IL‐23 were found in the supernatants of the CD11c^+^Gr‐1^+^ DCs, whereas only a slight and non‐significant increase in levels of IL‐1β, but not IL‐23, was seen in the supernatants of the CD11c^+^Gr‐1^−^ and CD11c^−^Gr‐1^+^ BMDC subsets.

### AR agonist promoted differentiation of Gr‐1^+^ DCs of LPS‐treated, but not untreated, naïve bone marrow cells

**Figure 6:**
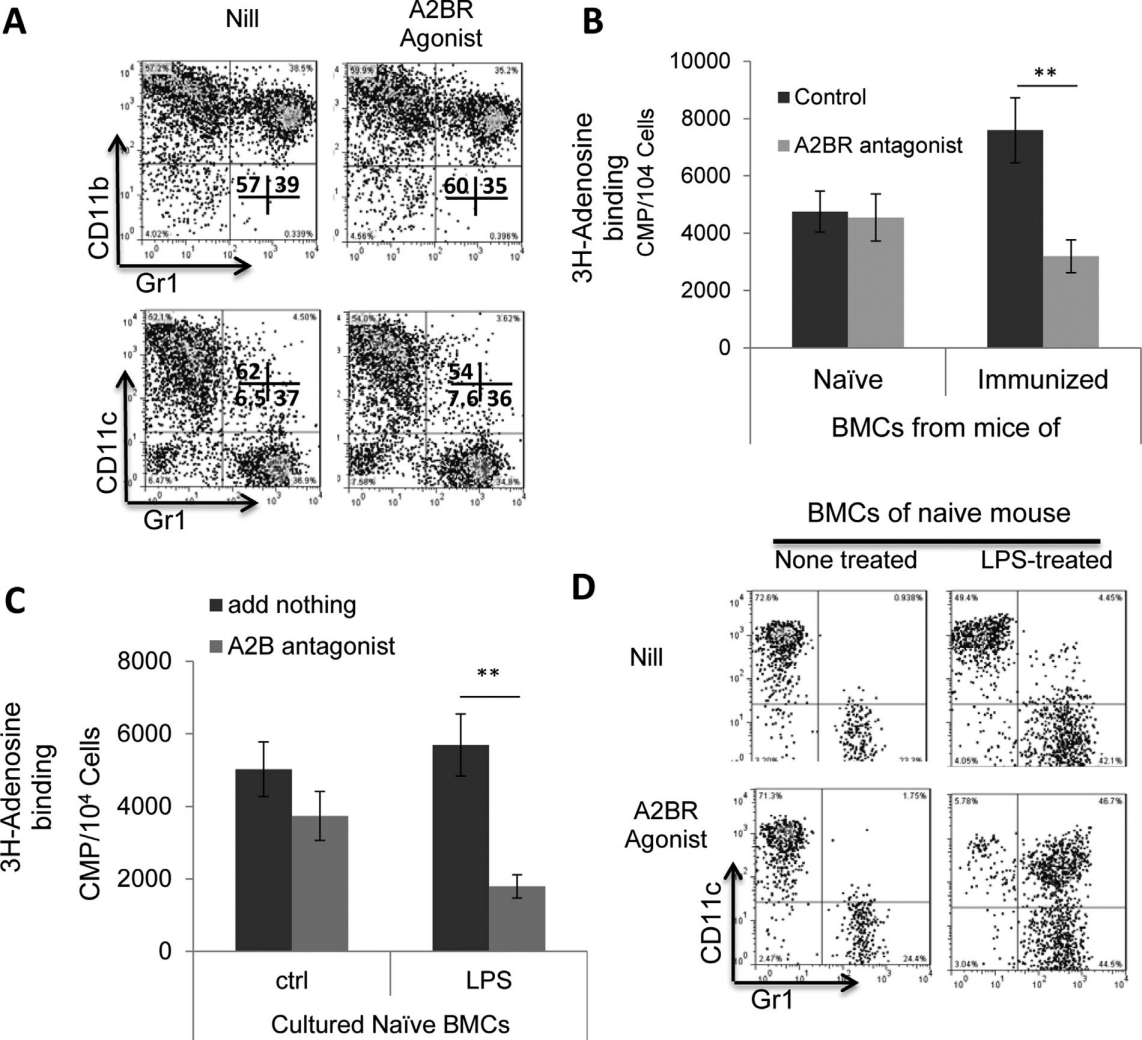
Naïve bone marrow cells were less sensitive to the agonist, but became increasingly sensitive after exposure to LPS. A: Naïve bone marrow cells were incapable of differentiating into CD11c^+^Gr‐1^+^ cells upon A2BR stimulation. Bone marrow cells of naïve B6 mouse were cultured for 5 days in medium continuing GM‐CSF (10 ng/mL) in the absence or presence of the A2BR agonist BAY 60‐6538 (100 nM). B: A2BR antagonist blocked adenosine binding to immunized, but not naïve, bone marrow cells. In 96‐well cell culture plates, 1 × 10^5^/mL bone marrow cells, obtained from either immunized or naïve mice, were incubated for 1 h with H^3^‐adenosine at final concentrations of 1.0 µM in triplicates, in the absence or presence of an A2BR antagonist MRS 1754 (100 nM). Cell‐bound and free H^3^‐adenosine was separated by harvesting the cells on a cell harvester (Perkin Elmer) and the cell‐associated radioactivity measured by liquid scintillation. C: After an exposure to LPS naïve bone marrow cells acquired increased adenosine binding activity via A2BR. Naïve bone marrow cells were tested for binding by H^3^‐adenosine, before (left panel) and after (right panel) an exposure to LPS (100 ng/mL), and in the absence or presence of the A2BR antagonist MRS 1754 (100 nM). D: A2BR agonist was more effective in stimulating CD11c^+^Gr‐1^+^ cells from LPS‐treated naïve bone marrow cells. Bone marrow cells of naïve B6 mouse were cultured for 5 days in medium continuing GM‐CSF (10 ng/mL), in the absence (upper panel) or presence (lower panel) of the A2BR agonist BAY 60‐6538 (100 nM), before (left panels) or after (right panels) LPS treatment.

Our results showed that the AR agonist was more effective promoting the generation of Gr‐1^+^ DCs from BMCs of immunized mouse, but was not as effective in naïve BMCs (Fig. 6A). Considering our previous observation that AR agonists only stimulated cytokine‐primed γδ T cells, but did not directly stimulate γδ T cells 27, we predicted that a prior exposure to stimulating factor of BMCs would enhance the agonist's effect. As demonstrated in Figure 6B, A2BR antagonist blocked the H^3^‐labeled adenosine binding to immunized BMCs more effectively than the naïve BMCs and this blocking effect of naïve BMCs became more apparent after the latter cells were pre‐exposed to LPS (Fig. 6C), suggesting that after stimulation the BMCs acquire increased ability to bind adenosine via A2BR and differentiate into Gr‐1^+^CD11c^+^ cells.

### CD11c^+^Gr‐1^+^ DCs express increased levels of CD25

**Figure 7:**
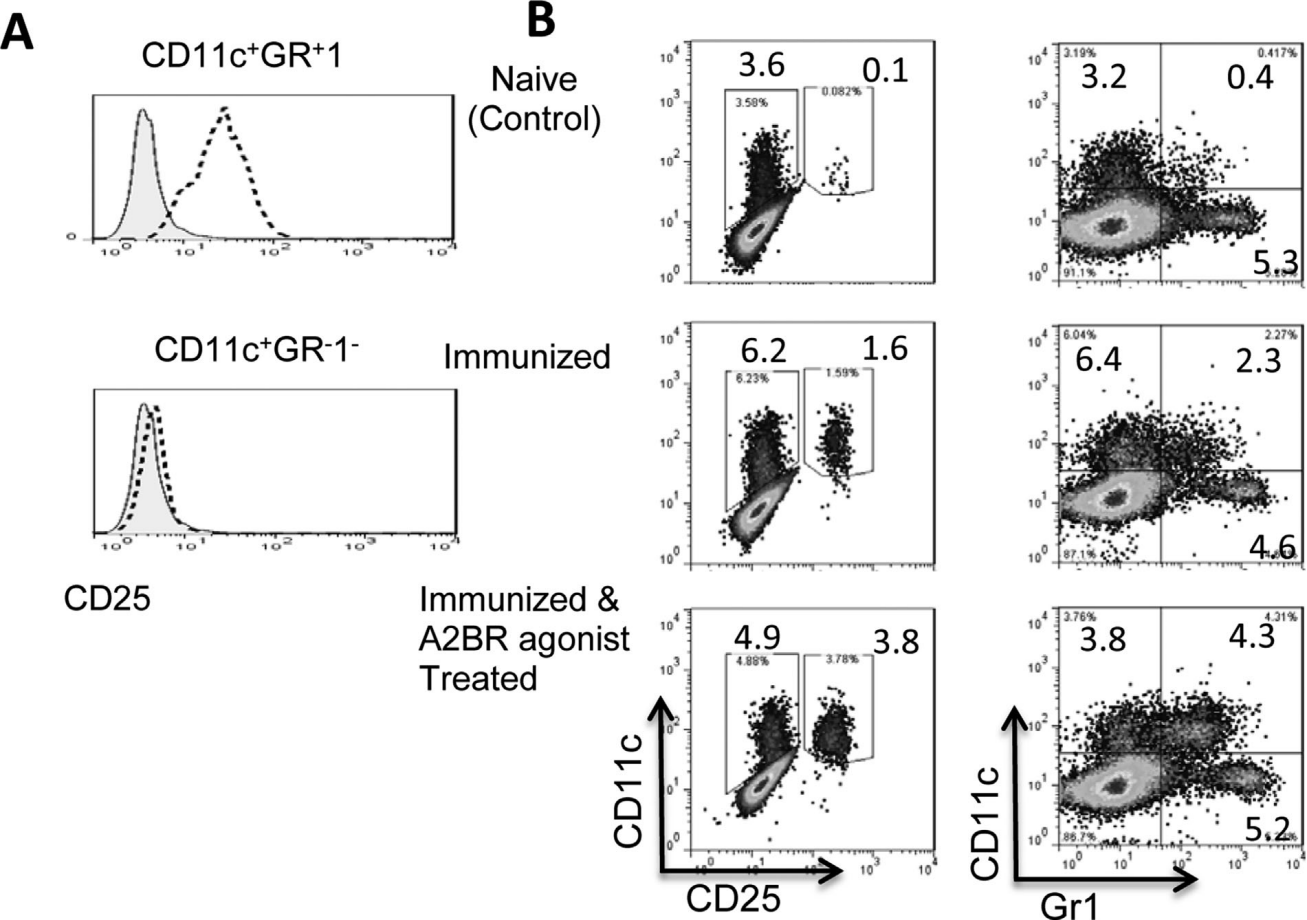
The Th17‐stimulatory effect of CD11c^+^ Gr‐1^+^ DCs is associated with increased CD25 expression. A: FACS staining of CD11c^+^Gr‐1^+^ and CD11c^+^Gr‐1^−^ cells after surface staining with a mAb specific for mouse CD25 (bold dotted lines). The shaded peaks represent the same cells stained with an isotype‐match control antibody. B: CD11c^+^Gr‐1^+^ and CD11c^+^ CD25^+^ splenic DC subsets are significantly increased in A2BR agonist (BAY 60‐6538)‐injected immunized B6 mice. B6 mice were left untreated (Naïve control) or were immunized with IRBP_1–20_ with or without simultaneous intraperitoneal injection of the A2BR agonist BAY 60‐6538 (1 m/kg), then, 13 days later, splenic cells were prepared and double‐stained with anti‐CD11c and anti‐Gr‐1 (left panels) or anti‐CD11c and anti‐CD25 (right panels) and examined by FACS.

We have previously reported that the CD25^+^ splenic DCs are more effective than the CD25^−^ subset in stimulating Th17 autoreactive T cells and γδ T cells 36, 37. We, therefore, examined whether the different Th17 stimulatory effect of CD11c^+^Gr‐1^−^ and CD11c^+^Gr‐1^+^ DCs correlated with their CD25 expression. As shown in Figure 7A, CD11c^+^Gr‐1^+^ cells expressed high levels of CD25, whereas CD11c^+^Gr‐1^−^ DCs expressed minimal amounts. We also examined whether i.p. injection of the A2BR agonist BAY 60‐6538 (1 mg/kg) into B6 mice on the same day as immunization would induce an increased number of CD25^+^ or Gr‐1^+^ DCs in the peripheral lymphoid organs (spleen). As shown in Figure 7B (top panels), only a few splenic CD11c^+^ DCs in naïve mice expressed Gr‐1 or CD25, but these values increased significantly in immunized mice examined 13 days after immunization (center panels) and, in the immunized mice injected with prior BAY 60‐6538, CD11c^+^Gr‐1^+^ and CD11c^+^CD25^+^ became the major populations in the CD11c^+^ splenic DCs. Functional tests confirmed that splenic CD11c^+^Gr‐1^+^ have a greater stimulatory effect on IL‐17^+^ autoreactive T cells (data not shown).

## Discussion

Although there is little disagreement that Th17 cells play a major role in the pathogenesis of diseases, including autoimmune diseases 38, 39, 40, 41, 42, the mechanism that promotes the activation and expansion of Th17 pathogenic T cells during autoimmune responses remained unclear and the inflammatory molecules that are capable of enhancing the function of Th17 autoreactive T cells remained to be identified. Such knowledge should improve our understanding of disease pathogenesis and provide insights for therapeutic intervention. Given our previous findings that an increased Th17 response is associated with increased γδ T cell activation 30, 32, 37 and with increased activation of a DC subset co‐expressing CD25 36, we were interested in identifying the pathogenic factors that cause γδ T cell activation and CD25^+^ DC expansion. In this study, we examined the effect of AR activators, because of previous findings that adenosine is involved in immune cell differentiation, inflammation, and immune responses 25, 43 and our previous finding that an AR agonist has an enhancing effect on the Th17 autoimmune response 27, 33. Since AR‐based treatments have been advocated for therapeutic use 44, 45, 46, 47, 48, clarification of the mechanisms by which an AR agonist enhances or inhibits an immune response should improve our understanding of disease pathogenesis and allow better pharmacological use of AR antagonists/antagonists.

In this study, we examined whether the effect of an AR agonist on DCs contributed to its inhibitory effect on Th1 responses and enhancing effect on Th17 responses. Our results showed that the non‐selective AR agonist NECA and the specific A2BR agonist BAY 60‐6538 skewed BMDC differentiation toward a distinct cell population co‐expressing CD11c and Gr‐1 (CD11c^+^Gr‐1^+^) (Fig. 2A, B) and that only an A2BR antagonist blocked the effect of NECA (Fig. 2C), showing that the A2BR was responsible for this effect. Interestingly, these CD11c^+^Gr‐1^+^ DCs had a strong stimulating effect on Th17 autoreactive T cells, in contrast to conventional CD11c^+^Gr‐1^−^ DCs cultured in the absence of an AR agonist, which preferentially stimulated Th1 autoreactive T cells. In consistent with our previously finding that γδ T cells are necessary for mediating Th17 enhancing function of CD25^+^ DCs 36, 37, we repeatedly observed that (Fig. 4) the Th17 promoting effect of CD11c^+^Gr‐1^+^ DC was dependent on the presence of γδ T cells and significantly increased productions of IL‐1β and IL‐23, two major cytokines driving Th17 response, were uniquely observed in the co‐culture of γδ, but not αβ, T cells and CD11c^+^Gr‐1^+^ DCs. Given our previous finding that an increased Th17 response is accompanied by increased expansion of CD25^+^ DCs 36, 37, we also examined whether the Th17‐stimulating CD11c^+^Gr‐1^+^ DC subset co‐expressed CD25 and found that CD11c^+^Gr‐1^+^ DCs expressed significantly higher levels of CD25 than conventional CD11c^+^Gr‐1^−^ DCs (Fig. 7A). We also found an increased number of splenic DCs expressing CD25 in immunized mice after a single injection of the A2BR‐specific agonist BAY 60‐6538 (Fig. 7B). Together, these results demonstrate that an A2BR agonist promotes a Th17 response in vivo and in vitro and that this effect involves enhanced differentiation of a strong Th17‐stimulating DC subset. Our observation supports previous findings that AR agonists enhance Th17 differentiation 8, 49, 50, 51 and have an effect on myeloid cell differentiation 11, 15, 52, 53. We have noticed that AR agonist does not have a strong stimulatory effect to naïve bone marrow cells, as opposed to its effect on immunized bone marrow cells; however, after an exposure to LPS, the naïve bone marrow cells acquired increased sensitivity to the agonist. This appears to be not unusual, given previous observations that A2BRs remained silent under normal physiological conditions but became active in pathological conditions 54 and the expression of A2BR was significantly upregulated during inflammation 14, 49. We have observed that adenosine binding of naive myeloid cells is mainly via non‐A2BR ARs. The expression pattern and binding affinity of all those four ARs were modulated by inflammatory environment. Thus, in immunized BMCs, or TLR ligand‐treated, naive BMCs, the binding activity of A2BR on myeloid cells increases significantly (data not shown).

Using a mouse EAU model, we recently found that injection of an A2AR agonist can have either an inhibitory or enhancing effect on Th17 responses, depending on when it was injected in relation to disease induction or the inflammatory status of the recipient mice 33. We then examined whether the A2BR agonist BAY 60‐6538 had a similar effect and our results showed that this was not the case, as it had an enhancing effect on the Th17 response, regardless of when it was administered (data not shown).

Accumulation of myeloid cells co‐expressing CD11b and Gr‐1, designated as myeloid‐derived suppressor cells (MDSCs) 55, 56, is seen in inflammatory sites and these cells are immune‐suppressive 55, 57, 58. Our results in the present study showed that Gr‐1^+^ cells in the agonist‐induced BMDCs were composed of two populations that expressed or did not express CD11c (Fig. 2B), and that the CD11c^−^Gr‐1^+^ cells had a suppressive effect on both Th1 and Th17 autoreactive T cell responses, while the CD11c^+^Gr‐1^+^ cells had a suppressive effect only on Th1 responder T cells (Fig. 3E), suggesting that Th17 and Th1 responses are regulated by different Gr‐1^+^ myeloid subsets.

We have previously reported that the enhanced Th17 response in EAU is associated with increased numbers of a DC subset expressing CD25 36, 37. The results of the present study supported this notion by showing that the appearance of increased numbers of splenic CD25^+^ DCs was associated with an enhanced Th17 response. Only a few CD25^+^ DCs were found in splenic DCs from a naïve mouse, but this number was significantly higher in immunized mice and again higher if the immunized mice were also injected with an A2BR agonist (Fig. 7B), and these effects were closely associated with increased Th17 responses in the recipient mice. Our future studies will be aimed at determining whether blockade of AR activation on DCs has an effect on expansion of CD25^+^ DCs and thus decreases the Th17 response and whether blockade of the γδ T cell‐DC interaction is effective in preventing pathogenesis mediated by Th17 autoreactive T cells. Such studies should advance our understanding of Th17 cell pathogenesis and improve therapeutic intervention.

## Conflict of Interest

None declared.
